# Rational Design of N-Doped Carbon Aerogel with Well-Defined Micropore Structure to Adsorb Dye from Water for High-Performance Lithium-Ion Battery Cathodes

**DOI:** 10.3390/gels11110857

**Published:** 2025-10-27

**Authors:** Yuang Xiong, Kelin Zhu, Lixia Yang, Rong Huang, Xingtang Liang, Binbin Zhang, Yanzhen Yin, Xia Chen, Zirun Chen

**Affiliations:** 1 Guangxi Key Laboratory of Green Chemical Materials and Safety Technology, Beibu Gulf University, Qinzhou 535011, China; 2 Guangxi Key Laboratory of Beibu Gulf Marine Biodiversity Conservation, College of Marine Sciences, Beibu Gulf Ocean Development Research Center, Beibu Gulf University, Qinzhou 535011, China

**Keywords:** carbon aerogels, dye adsorption, lithium-ion batteries, cathode materials, resource utilization

## Abstract

N-doped carbon aerogels have garnered increasing research interest in the field of energy and environment due to their unique structural features. Organic dyes, which contain redox-active sites and act as pollutants, are attractive candidates for cathode materials in Li-ion batteries but still suffer from poor cycle stability and rate performance. Therefore, there is still a lack of an easy and effective approach to rationally design the pore structure of N-doped carbon aerogels for efficiently and stably trapping dye molecules and converting them into high-performance cathode materials. Herein, we propose an innovative strategy for preparing nitrogen-doped carbon aerogels with a well-defined micropore structure (MNCAs) for efficient adsorption of dye molecules, subsequently converting them into high-performance lithium-ion battery cathode materials. MNCAs were synthesized via Schiff-based polymerization using polyhedral oligomeric silsesquioxane (POSS) as a template, resulting in a carbon framework with well-defined micropores. Benefiting from their high specific surface area and well-defined micropore structure, MNCAs exhibited a maximum adsorption capacity at equilibrium of 2273 mg g^−1^ for indigo. Notably, the indigo@nitrogen-doped carbon aerogel composite (IDG@MNCAs) exhibits high specific capacity, outstanding cycling stability, and remarkable rate capability. The discharge specific capacity of IDG@MNCAs retains 89% of its capacity (120 mAh g^−1^) after 200 cycles at 100 mA g^−1^ and maintains 70% capacity retention after 1200 cycles at the higher current density of 1000 mA g^−1^, surpassing many recently reported organic cathode materials.

## 1. Introduction

With the rapid development of society, the global energy crisis and environmental pollution have become critical issues due to the increasing depletion of fossil fuels and their extensive use. To address these challenges, researchers are exploring numerous solutions in the field of energy conversion and storage [1,2,3] and water purification [4,5,6], separately. For energy conversion and storage, as one type of efficient green energy technology, rechargeable Li-ion batteries have been commercialized and extensively studied [7,8,9,10]. However, they still suffer from high cost, complex processing, and serious environment pollution during the production of cathodes. As for the environment pollution, organic pollutants (especially organic dye) are harmful to both human health and ecosystems due to their toxicity and persistence, which are resistant to natural breakdown processes [11,12,13]. To date, physical adsorption is considered an efficient method for the effective removal of organic dyes from water [14,15]. Nevertheless, the high cost and complex recycling processing hinder its practical application potential. Therefore, converting organic dyes/adsorbent composites into high-value-added products remains a challenge.

It is worth noting that developing organic cathode materials for rechargeable Li-ion batteries would be a promising strategy for addressing these two problems simultaneously. To date, numerous organic molecules containing redox groups have been investigated as potential cathode materials for Li-ion batteries [16,17]. Among them, organic molecules with carbonyl groups stand out as the most promising candidates for cathode materials, owing to their high theoretical specific capacities and fast electrochemical reaction kinetics [18,19,20,21]. However, the low conductivity and dissolution of electrolytes of organic molecules hinder their application in high-performance cathode materials. For instance, 2,6-dimethoxy-9,10-anthraquinone possesses high initial specific capacity (196 mAh g^−1^ at 40 mA g^−1^) but poor cycle stability (104 mAh g^−1^ after 80 cycles, 53.1% retention) and an inferior rate capacity (75 mAh g^−1^ at 1000 mA g^−1^) [22]. In this way, it becomes imperative to devise efficient adsorbents that are capable of capturing carbonyl-based organic dyes from water and transforming them into high-performance cathode materials.

N-doped carbon aerogels, a subclass of porous carbon materials, have emerged as promising candidates for addressing both energy storage and environmental remediation challenges due to their unique physical and chemical properties [23]. N-doped carbon aerogels are characterized by their ultra-low density, high specific surface area, tunable pore structure, and excellent electrical conductivity, which make them ideal for applications in water purification [24,25] and electrochemical energy storage [26]. Specifically, their three-dimensional interconnected framework facilitates rapid mass transfer and ion diffusion, while beneficial N doping provides abundant active sites for adsorption and redox reactions. Thus, N-doped carbon aerogels are suitable adsorbents for carbonyl-based organic dyes, and most importantly, they can also serve as the conductive framework in organic cathodes. Despite these advantages, there is still a challenge that needs to be addressed: most N-doped carbon aerogels lack a well-defined micropore structure to trap dye molecules and prevent their dissolution into the electrolyte during the electrochemical reaction process. Therefore, it is necessary to develop a facile and efficient strategy to fabricate novel carbon aerogels with well-defined micropore structures and abundant active sites for the simultaneous removal of carbonyl-based organic dyes from water and the fabrication of high-performance organic cathode materials.

Herein, to address the aforementioned challenges in carbon aerogels, we propose a novel synthetic strategy for the preparation of carbon aerogels with high specific surface area, well-defined pore structure, and beneficial N doping. Central to this approach is the rational design of monomers and solvents for Schiff-based polymerization, which enables precise control over the pore structure and gelation time of the resulting carbon aerogels. Specifically, the introduction of polyhedral oligomeric silsesquioxane (POSS) into the polymerization framework generates a homogeneous organic/inorganic molecular interface, resulting in well-defined micropores in the carbon framework after pyrolysis and acid etching. To demonstrate the general applicability of our approach, we selected two representative carbonyl-based dyes, indigo and alizarin red, which are commonly found in industrial effluents. Specifically, the carbon aerogel demonstrated efficient adsorption of carbonyl-based organic dyes (indigo and alizarin red), and importantly, the resulting indigo/carbon aerogel composites displayed outstanding electrochemical performance as a cathode material in lithium-ion batteries (LIBs). This is the first time that indigo has been employed for energy storage in rechargeable batteries, and it exhibits remarkable specific capacity, cyclic stability, and rate capability. This work not only provides new insights into the treatment and resource utilization of dye from water but also promotes the development of high-performance lithium-ion battery cathode materials.

## 2. Results and Discussion

**Scheme 1:**
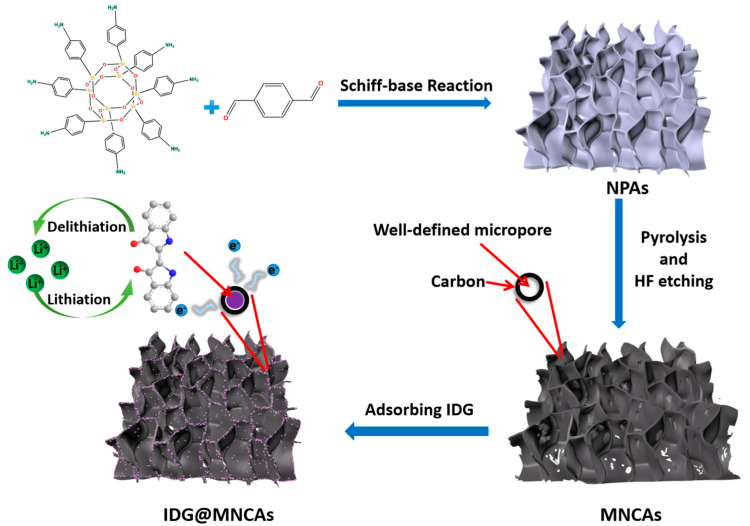
Schematic illustration of the synthesis of MNCAs and IDG@MNCAs materials.

The fabrication process of N-doped carbon aerogels with well-defined micropore structures (MNCAs) is schematically depicted in Scheme 1. Initially, N-doped polymer aerogels (NPAs) are synthesized via the polycondensation of POSS-NH_2_ and terephthalaldehyde (TPA) in 1,4-dioxane, facilitated by acetic acid as a catalyst at ambient temperature, followed by freeze-drying. During this reaction, imine bonds form between each POSS-NH_2_ and TPA molecule, resulting in a robust three-dimensional crosslinked network. The as-prepared NPAs integrate carbon precursors and well-defined micropore-scale silica templates, eliminating the need for complex casting and interfacial engineering steps typically required in conventional templating methods. The homogeneous carbon/silica interface within the NPAs ensures the formation of highly dispersed well-defined micropore-scale silica in the carbonized NPAs (cNPAs). Subsequent etching of these silica templates yields the final MNCAs. The as-prepared MNCAs can efficiently adsorb indigo and convert it into high-performance cathode materials for Li-ion batteries.

**Figure 1:**
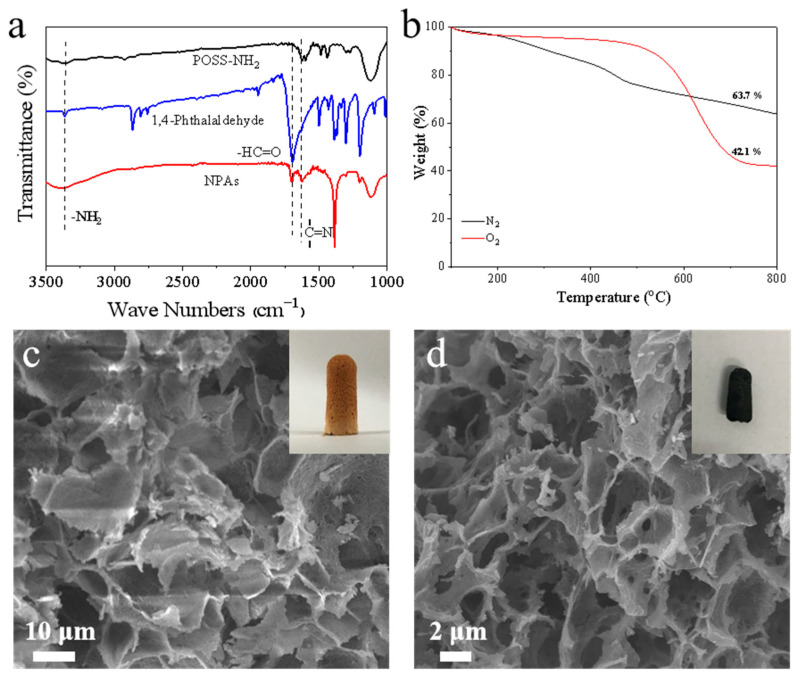
(**a**) FTIR spectrum of NPAs. (**b**) TGA curves of NPAs under N_2_ and O_2_ stream. SEM image of (**c**) NPAs and (**d**) cNPAs.

In order to acquire the structure features of NPAs, Fourier transform infrared (FTIR) spectroscopy and Raman spectrum analysis were carried out. The FTIR spectrum in Figure 1a reveals that the absorbing peak located at 1623 cm^−1^ can be assigned to the C=N groups, which suggests the occurrence of Schiff-base polymerization between POSS-NH_2_ and TPA. Moreover, the C=O and -NH_2_ groups, situated at 1699 and 3370 cm^−1^, respectively, are associated with the presence of the unreacted aldehyde and amino groups within the NPAs [27]. Moreover, the Raman spectra of the NPAs show two characteristic peaks at 1573 and 1623 cm^−1^, associated with the C=C and C=N groups, respectively. These results confirm the successful preparation of NPAs (Figure S1).

The choice of catalyst and solvent significantly influences the sol–gel reaction kinetics. Specifically, the addition of 1 mL of acetic acid catalyst reduces the gelation time from 30 min (without acetic acid) to merely 2 s (Video S1). Furthermore, altering the solvent to DMF and 1,2-dichloroethane results in the formation of a bulk structure and spherical structure (Figure S2), which is attributed to variations in solvent–polymer interactions, such as differences in polarity. Our rapid gelation synthesis strategy is conducive to high-throughput and scalable production of MNCAs.

**Figure 2:**
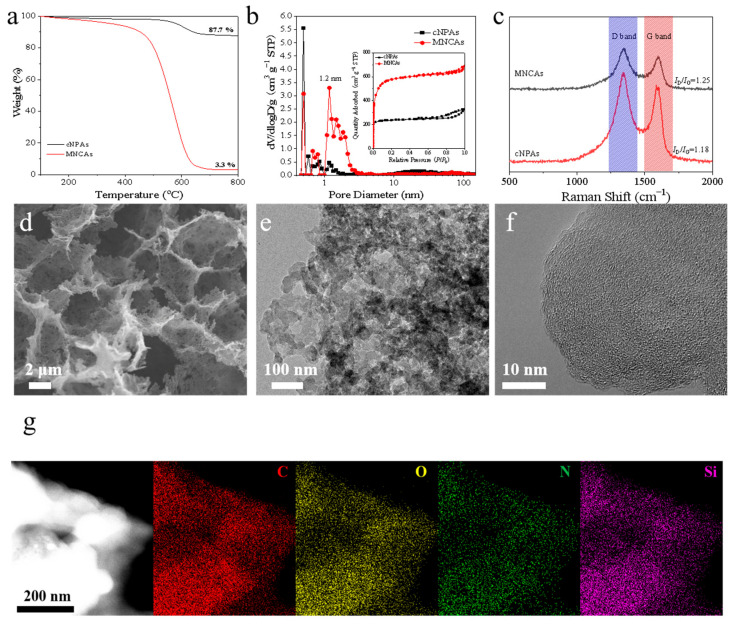
(**a**) TGA curves of cNPAs and MNCAs. (**b**) Pore size distribution and N_2_ adsorption–desorption isotherms (insert) of cNPAs and MNCAs. (**c**) Raman spectra of cNPAs and MNCAs. (**d**) SEM image of MNCAs. (**e**,**f**) HRTEM images of MNCAs. (**g**) HAADF-STEM and elemental mapping of cNPAs.

The morphological characteristics of the related samples were analyzed using an electron microscope. Scanning electron microscopy (SEM) (Figure 1c) reveals that the NPAs exhibit a three-dimensional interconnected macroporous architecture. The robust organic–inorganic network endows the NPAs with exceptional thermostability, as evidenced by the high carbon residue yield observed in thermogravimetric analysis (Figure 1b). This property ensures successful thermal conversion of the NPAs into carbonaceous frameworks while preserving their architecture integrity. SEM images of the cNPAs (Figure 1d) confirm that the original polymer architecture is retained after carbonization, resulting in three-dimensional interconnected carbon/silica composites. Transmission electron microscopy (TEM) images (Figure S3) further demonstrate the absence of silica agglomeration, attributed to the homogeneous aromatic–silica hybrid interface. Elemental mapping (Figure 2g) reveals a uniform distribution of carbon (C), nitrogen (N), silicon (Si), and oxygen (O) elements throughout the cNPAs skeletons.

The three-dimensional macroporous structure facilitates complete removal of the well-defined micropore-scale silica template via hydrofluoric acid (HF) etching, yielding a well-defined micropore carbon framework. The removal efficiency of the silica template was assessed using thermogravimetric analysis (TGA) conducted under an oxygen stream. As shown in Figure 2a, the silica content was drastically reduced from 87.7 wt% (cNPAs) to 3.3 wt% (MNCAs), revealing that the tremendous well-defined micropores can be formed from the nanosilica template after HF etching. Moreover, the resulting MNCAs retain the original shape of the NPAs (Figure 1d and Figure 2d). HRTEM images (Figure 2e,f) indicate that the carbon skeleton of the MNCAs consists of turbostratic carbon nanodomains, characterized by disordered stacking of graphene layers. This carbon skeleton enhances surface area and pore accessibility, making the MNCAs suitable for applications requiring high adsorption capacity and electrochemical performance.

The pore structure of the MNCAs was investigated using nitrogen (N_2_) adsorption–desorption measurements. As depicted in Figure 2b, the MNCAs exhibit a steep rise in the nitrogen adsorption stage at low relative pressures and no hysteresis loops at higher relative pressures, indicating a predominantly micropore structure within the carbon framework. The Brunauer–Emmett–Teller (BET) surface area (S_BET_) and total pore volume of the MNCAs were determined to be 1986 m^2^ g^−1^ and 1.06 cm^3^ g^−1^, respectively. Using the t-plot method, the micropore area of the MNCAs was calculated to be 1261 m^2^ g^−1^, corresponding to a micropore ratio of 63%. This result confirms that the high surface area of the MNCAs is predominantly attributed to the abundance of micropores in their carbon skeleton. In contrast, cNPAs without removal of the silica template exhibit a much lower BET surface area of 789 m^2^ g^−1^ and a pore volume of 0.51 cm^3^ g^−1^, highlighting that the micropore structure in the MNCAs originates primarily from the silica templates.

The pore size distribution curve of the MNCAs (Figure 2b) reveals a narrow distribution, with well-defined micropores centered at approximately 1.2 nm [25,28]. This pore size is close to the diameter of the POSS molecules, further corroborating the role of the well-defined micropore-scale silica templates in creating these micropores. This structural design not only maximizes surface area but also ensures precise control over pore size distribution, making the MNCAs highly suitable for applications requiring high surface area and tailored pore structure.

The structure and chemical elemental compositions of the MNCAs were further analyzed using X-ray powder diffraction (XRD), Raman spectroscopy, and X-ray photoelectron spectroscopy (XPS). XRD measurements (Figure S4) reveal two broad diffraction peaks at 23° and 44°, indicators of limited graphitization within the carbon framework. Raman spectroscopy (Figure 2c) confirms the coexistence of graphitic and disordered carbon structures, with characteristic peaks at approximately 1347 cm^−1^ (D band) and 1598 cm^−1^ (G band). Additionally, the higher *I*_D_/*I*_G_ ratio of the MNCAs compared to cNPAs suggests an increase in vacancies and defect sites, which is caused by the removal of silica templates leading to an increase in micropores. XPS analysis (Figure S5) reveals that the MNCAs are primarily composed of carbon (C), nitrogen (N), silica (Si), and oxygen (O), with N and O contents of 1.69 at% and 9.17 at%, respectively (Table S1). The presence of heteroatoms with distinct electronegativities, such as N and O, modulates the surface chemistry of the carbon framework and enhances its adsorption performance through synergistic effects [29]. High-resolution N 1s spectra (Figure S6) identify four types of nitrogen-containing functional groups: pyridinic N (398.6 eV), pyrrolic N (400.2 eV), graphitic N (401.4 eV), and oxidized N (403.3 eV). High-resolution C 1s and O 1s spectra (Figure S6) further elucidate the distribution of carbon-nitrogen and carbon-oxygen species, which influence the local charge distribution of carbon atoms and enhance their affinity toward pollutants [30].

To evaluate the general applicability of this strategy, different monomers were used to synthesize the MNCAs. As shown in Figure S7, altering the dialdehyde from TPA to glyoxal (MNCAs-g) and 4,4″-p-Terphenyldicarboxaldehyde (MNCAs-t) resulted in a three-dimensional interconnected framework, indicating the effectiveness of this synthetic strategy. Furthermore, the S_BET_ of MNCAs-g and MNCAs-t are 847 and 1914 m^2^ g^−1^, respectively (Table S2). The micropore size of MNCAs-g and MNCAs-t are centered at 1.2 nm, approximately the size of the POSS molecule (Figure S8). These results indicate the effectiveness of our synthetic strategy to obtain N-doped carbon aerogel with well-defined micropores.

**Figure 3:**
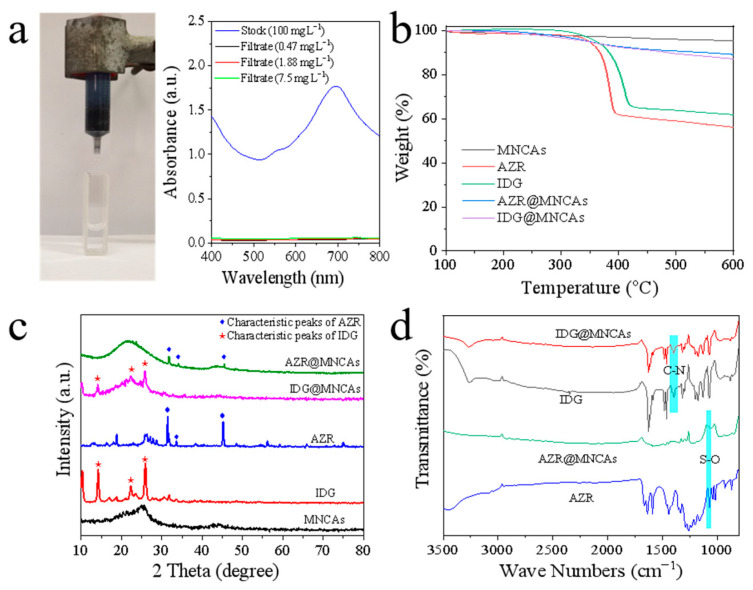
(**a**) Photograph of flow through adsorption experiment and UV–vis spectra of IDG at different flow rates. (**b**) TGA curves of different samples under N_2_ stream. (**c**) XRD patterns of different samples. (**d**) FTIR spectra of different samples.

Dye adsorption is achieved by immersing the MNCAs in artificial effluent containing indigo (IDG) or alizarin red (AZR), which are representative carbonyl-based organic dyes. This straightforward method demonstrates significant adsorption capacity (Figure S9). Additionally, nearly 99% of IDG can be eliminated from water after the adsorbate solution swiftly passes through an MNCA-packed column, revealing strong potential for industry applications (Figure 3a). The adsorption isotherm of MNCAs toward IDG is acquired by fixing the MNCA concentration at 0.6 mg mL^−1^ and elevating the IDG concentration in the range from 100 to 600 mg L^−1^ (Figure S10). The results suggest effective removal efficiencies of MNCAs even at higher pollutant concentrations. Based on the Langmuir fit, the maximum adsorption capacity at equilibrium (*q*_max_) of MNCAs is 2273 mg g^−1^. The adsorption capacity in different conditions (pH, temperature, and NaCl concentrations) was further evaluated (Figure S11). Temperature and NaCl concentration had an insignificant impact on the removal efficiency of IDG. However, the uptake of IDG decreased with increasing pH values, which can be attributed to the enhanced electrostatic repulsion between IDG and MNCAs under high pH. Thermogravimetric analysis (TGA) data indicate that the loading percentages of IDG and AZR in the composites are 25.3% and 18.0%, respectively (Figure 3b). The successful adsorption of dye molecules onto the MNCAs is further evidenced by XPS and XRD. The XPS survey spectrum reveals an increased N content (6.53 at%) in IDG@MNCAs and the presence of an additional S element in AZR@MNCAs compared to MNCAs, indicating the successful introduction of IDG and AZR into MNCAs (Figure S5). Furthermore, XRD patterns (Figure 3c) reveal that the weak characteristic diffraction peaks of IDG and AZR appear in IDG@MNCAs and AZR@MNCAs, which are consistent with the results of XPS. Compared to the FTIR spectra of indigo and alizarin red, the symmetric stretch of C-N at 1392 cm^−1^ (attributed to IDG) and the asymmetric stretch of S-O at 1068 cm^−1^ (assigned to AZR) exhibit slight shifts in their corresponding dye@MNCAs composites, demonstrating strong molecular interactions between the dyes and the MNCAs (Figure 3d) [31]. Additionally, a blue shift of the G band and a decrease in the *I*_D_/*I*_G_ ratio in the Raman spectra of both IDG@MNCAs and AZR@MNCAs reveal molecular interactions between the dye molecules and the carbon aerogel matrix (Figure S12).

**Figure 4:**
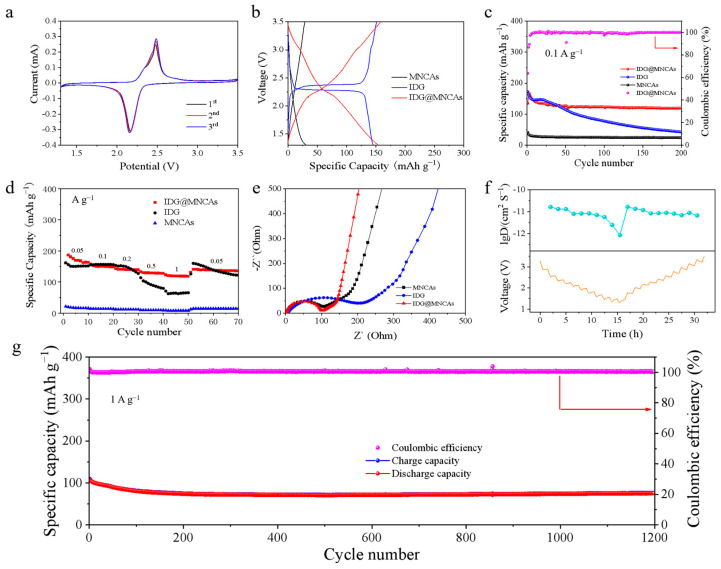
(**a**) CV curves of IDG@MNCA electrode at a scan rate of 1 mV s^−1^. (**b**) Galvanostatic discharge–charge curves and (**c**) cycling performance of different electrodes tested at 100 mA g^−1^. (**d**) Rate capacity test of different electrodes under different current densities. (**e**) Nyquist plots of different electrodes after 100 cycles at 100 mA g^−1^. (**f**) GITT curve and diffusion coefficient of the IDG@MNCAs electrode. (**g**) Long cycling stability of IDG@MNCAs at 1000 mA g^−1^.

The electrochemical performance of IDG@MNCAs as cathodes for lithium-ion batteries (LIBs) was evaluated using traditional 2032 coin-type cells with pure Li metal foils as the anodes. For comparison, MNCAs and IDG were also tested under the same conditions. Cyclic voltammogram (CV) curves of different electrodes tested at 1 mV s^−1^ in the voltage range of 1.3 to 3.5 V (vs. Li/Li^+^) are presented in Figure 4a. Evidently, IDG@MNCAs exhibit significantly improved gravimetric current compared to that of IDG, indicating the enhanced electronic conductivity and electrolyte accessibility after embedding of IDG into MNCAs (Figure S13). Highly repeatable redox peaks at around 2.16 V and 2.49 V correspond to the redox reactions of IDG molecules, which agree well with the corresponding galvanostatic charge/discharge profile (Figure 4b).

The galvanostatic charge/discharge tests of all electrodes were conducted at a current density of 100 mA g^−1^ for 200 cycles (Figure 4c). As anticipated, pristine carbon aerogel exhibited poor Li storage behavior, characterized by low specific capacity. The initial discharge capacity was merely 42 mAh g^−1^ and further rapidly decreased to 24 mAh g^−1^ after 200 cycles. Moreover, IDG displays high initial specific capacity (172 mAh g^−1^) but poor cycle stability (43 mAh g^−1^ after 200 cycles) due to the low conductivity and dissolution in the electrolyte. In stark contrast, the Li storage capacity and cyclic stability of IDG@MNCAs were significantly enhanced upon immobilization of IDG on MNCAs (Figure S14). The initial discharge capacity of the IDG@MNCAs was 135 mAh g^−1^ and stayed at approximately 120 mAh g^−1^ after 200 cycles, indicating the high specific capacity retention of IDG@MNCAs (89%) compared to that of IDG (25%). Specifically, the IDG@MNCA cathode retained a reversible capacity of 75.5 mAh g^−1^ after 1200 cycles at a high current density of 1000 mA g^−1^, corresponding to a high capacity retention ratio of 70% and a very small capacity decay of 0.025% per cycle (2.5% per 100 cycles), indicating its long-term stability at high current density (Figure 4g). These values surpass those of previously reported organic cathodes and even some organic/carbon hybrid composite cathodes (Table S3) [32,33,34,35,36,37,38,39].

To further evaluate the rate capability of the electrodes, tests were performed under programmed current densities ranging from 50 to 1000 mA g^−1^ (Figure 4d). Notably, the IDG@MNCAs electrode demonstrated the best rate performance among the tested electrodes (MNCAs and IDG). This is likely related to its conductive carbon skeleton of carbon aerogel and the stable implant of IDG into MNCAs. The capacities of the IDG@MNCA electrode at current densities of 50, 100, 200, 500, and 1000 mA g^−1^ were approximately 147, 142, 134, 124, and 112 mAh g^−1^, respectively. Remarkably, when the current density was reverted to 50 mA g^−1^ after testing at various densities, the capacity of the IDG@MNCA electrode gradually recovered to approximately 130 mAh g^−1^, indicating its outstanding rate capability and structural stability.

To investigate the charge transfer kinetics of the samples, electrochemical impedance spectroscopy (EIS) was performed over a frequency range spanning from 100 kHz to 0.1 Hz [40,41]. As shown in Figure S15, IDG@MNCA shows a larger charge transfer resistance (R_ct_) of 106.7 Ω before cycling and a decreased R_ct_ of 91.3 Ω after 100 cycles at 100 mA g^−1^. Furthermore, the R_ct_ of IDG@MNCAs (91.3 Ω) is much lower than that of IDG (162.1 Ω) and is comparable to that of MNCAs (82.5 Ω) (Figure 4e). This reduction in interfacial charge transfer resistance indicates enhanced electron transport kinetics and improved electrochemical reactivity, which are critical for achieving higher reversible capacity and superior rate capability. Notably, high specific surface area (1986 m^2^ g^−1^) and an optimal micropore architecture with an average pore diameter of approximately 1.2 nm and a 3D carbon framework ensures IDG@MNCAs have an extensive contact area with the electrolyte and hence facilitates ion diffusion and electrolyte penetration.

It is worth noting that the IDG molecule was used in the cathode materials for Li-ion batteries for the first time. In order to study the the Li^+^ diffusion coefficients of (D_Li^+^_) for IDG@MNCAs, galvanostatic intermittent titration technique (GITT) measurement was carried out (Figure 4f). Based on the GITT results, the apparent Li-ion diffusion coefficients (D_Li^+^_) were evaluated and found to be ~10^−11^ S cm^−1^, revealing the fast reaction kinetics of IDG@MNCAs during the Li insertion/ extraction process.

**Figure 5:**
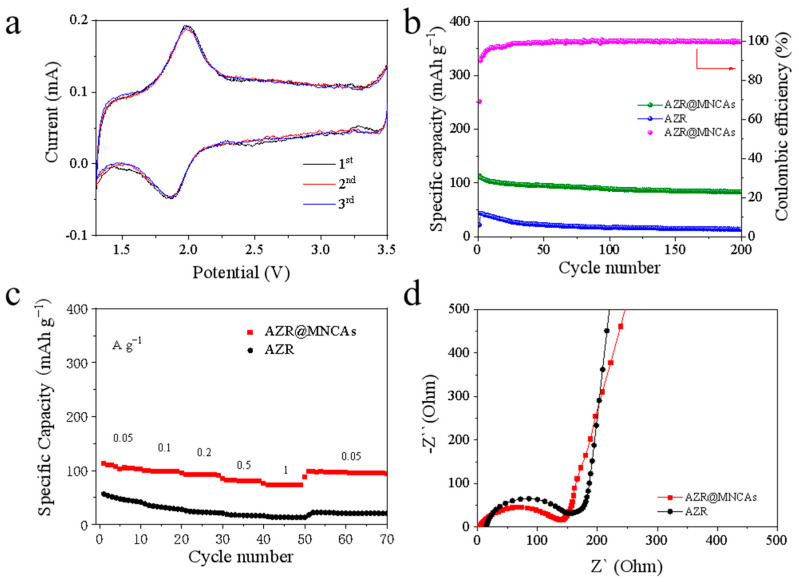
(**a**) CV curves at a scan rate of 1 mV s^−1^. (**b**) cycling performance of AZR and AZR@MNCAs tested at 100 mA g^−1^. (**c**) Rate capacity test of AZR and AZR@MNCAs under different current density. (**d**) Nyquist plots of AZR and AZR@MNCAs after 100 cycles at 100 mA g^−1^.

To expand the applicability of carbon aerogel composites in dye removal and energy storage applications, other dye@MNCAs such as AZR@MNCAs were synthesized by immersing the MNCA substrate into AZR solution. Cyclic voltammetry (CV) measurements (Figure 5a) exhibited distinct pairs of redox peaks centered at approximately 1.88 V (vs. Li/Li^+^), corresponding to the redox activities of AZR. After 200 charge/discharge cycles at a current density of 100 mA g^−1^, the AZR revealed a poor cycling performance (32%), which is the same shortcoming as IDG. In contrast, the AZR@MNCAs retained 76% of their initial capacity (Figure 5b), demonstrating remarkable cycle stability and structural stability. The AZR@MNCAs also exhibited exceptional rate capability, delivering a reversible capacity of 73.5 mAh g^−1^ even at a high current density of 1000 mA g^−1^ (Figure 5c). EIS curves (Figure 5d) further indicated that introducing AZR into MNCAs enhances electron transport kinetics and improves electrochemical reactivity. Water containing multiple dyes was further studied. For the AZR-IDG@MNCAs, the competition of adsorption sites between AZR and IDG changes the surface chemistry and electronic structure of MNCAs, leading to variation in electrochemical performance (Figure S16). The redox-active moieties of IDG and AZR serve as representative examples of carbonyl-based dyes. The introduction of MNCAs with a 3D carbon framework, high conductivity, and “nanoconfined effect” can greatly improve the electrochemical performance of IDG and AZR. Based on their effective utilization as cathode materials in LIBs, our approach can be extended to process and fabricate other electrochemically active dye molecules into active electrode materials, such as isatin, Methyl Orange, Alizarin green, OrangeI, and Oxonol Yellow K. This strategy not only addresses environmental concerns related to water purification but also offers a sustainable pathway for converting pollutants into valuable energy storage materials, thereby bridging environmental remediation and energy storage applications.

## 3. Conclusions

In this work, we successfully synthesized a type of N-doped carbon aerogel with a well-defined micropore structure for efficient dye adsorption, then we converted the resulting dye@MNCAs into high-performance cathode materials. By leveraging the “nanoconfined effect” and conductive properties of MNCAs, two representative classes of organic dyes (indigo and alizarin red) were efficiently adsorbed and resourcefully converted into high-performance cathode materials. The IDG@MNCAs demonstrated exceptional electrochemical performance, achieving a high specific capacity of 135 mAh g^−1^ at 100 mA g^−1^ and retaining 89% of its initial capacity after 200 cycles. This performance is attributed to the 3D carbon framework, high conductivity, and “nanoconfined effect” of MNCAs, which enhances Li^+^ ion diffusion, electrolyte accessibility, and most importantly, stable immobilization of redox-active dye molecules. The scalable strategy and exceptional high-performance Li storage make the dye@MNCAs promising candidates for sustainable energy storage applications. This work not only achieves efficient dye adsorption but also establishes a platform for the rational design of dye–carbon aerogel hybrid electrodes.

## 4. Materials and Methods

### 4.1. Materials

Octaaminophenyl polyhedral oligomeric silsesquioxane (POSS-NH_2_) were purchased from Bidepharm (Shanghai, China). Glyoxal, terephthalaldehyde, 4,4″-p-Terphenyldicarboxaldehyde, 1,4-dioxane, DMF, 1,2-dichloroethane, and hydrofluoric acid (HF) were purchased from Macklin (Shanghai, China). Acetic acid was acquired from Guangzhou Chemical Reagent Company (Guangzhou, China). All chemicals were directly used without further purification.

### 4.2. Sample Preparation

#### 4.2.1. Synthesis of N-Doped Carbon Aerogels with Well-Defined Micropore Structure (MNCAs)

To synthesize MNCAs, 0.4 mmol of TPA was dissolved in 1 mL of 1,4-dioxane to form a homogeneous solution. The solution was then mixed with 1 mL of 1,4-dioxane containing 0.1 mmol of POSS-NH_2_. After continuing reaction for approximately 30 min, the as-prepared polymer gel was freeze-dried for 48 h to obtain N-doped polymer aerogels (NPAs). Subsequently, the NPAs were carbonized at 900 °C for 3 h with a heating rate of 5 °C min^−1^ under a nitrogen atmosphere, resulting in carbonized N-doped Polymer Aerogels (cNPAs). The final MNCAs were obtained by etching the cNPAs with 10 mL of 20% hydrofluoric acid (HF) for 12 h, followed by washing with excess water and vacuum-drying at 60 °C.

For comparison, different morphologies of NPAs were prepared via polymerization in DMF or 1,2-dichloroethane. Moreover, MNCAs-g and MNCAs-t were synthesized using the same procedure, but changing TPA to Glyoxal and 4,4″-p-Terphenyldicarboxaldehyde, respectively.

#### 4.2.2. Synthesis of IDG@MNCAs

To prepare IDG@MNCAs, 20 mg of IDG were dispersed in 50 mL of deionized water and sonicated for 30 min to form IDG solution (400 mg L^−1^). Subsequently, 50 mg of MNCAs were added to the solution and stirred at room temperature for 12 h. The resulting products were collected, washed thoroughly with water, and vacuum-dried to obtain IDG@MNCAs. For comparison, AZR@MNCAs and AZR-IDG@MNCAs were synthesized under identical conditions.

### 4.3. Sample Characterization

The morphology of the samples was analyzed using field-emission scanning electron microscopy (FESEM, S-4800, Hitachi, Tokyo, Japan) and field-emission transmission electron microscopy (FETEM, Tecnai G2 F20 S-TWIN, FEI, Waltham, MA, USA). Chemical compositions were investigated via X-ray photoelectron spectroscopy (XPS, Thermo ESCALAB 250, Thermo Fisher Scientific, Waltham, MA, USA). Structural information was obtained from powder X-ray diffraction (XRD, Bruker D8ADVANCE, Bruker, Billerica, MA, USA, Cu Kα radiation, 10–80° scan range). Functional group analysis was performed using Fourier-transform infrared spectroscopy (FT-IR, PerkinElmer Frontier, PerkinElmer, Inc., Waltham, MA, USA, KBr pellet method). Thermogravimetric analysis (TGA, METTLER TOLEDO TGA/DSC, Greifensee, Switzerland) was conducted from 100 °C to 800 °C at a heating rate of 10 °C min^−1^ under oxygen or nitrogen atmospheres. Raman spectra were recorded using DXR Thermo Fisher Scientific with a 514 nm laser. Nitrogen adsorption–desorption isotherms and pore-size distribution were measured using a Micromeritics ASAP2020 analyzer (Micromeritics Instrument Corporation, Norcross, GA, USA). To assess pollutant removal efficiency, the filtrate was analyzed via UV–vis spectrophotometry (TU-1950, BEIJING PUXI GENERAL INSTRUMENT Co., Beijing, China).

### 4.4. Thermodynamic Analysis of Adsorption

In the adsorption experiments, 6 mg of MNCAs were added into 10 mL of pollutant solutions with varying concentrations (100, 200, 300, 400, 500, and 600 mg L^−1^) at pH 7. The suspension was stirred at room temperature for 1 h at a speed of 300 rpm to ensure adsorption equilibrium. Adsorption experiments at different temperatures, pH values, and NaCl concentrations were conducted under the same conditions.

The maximum adsorption capacity was determined using the Langmuir adsorption isotherm model, which is described by the following equation:

(1) $$\frac{1}{q_{e}}=\frac{1}{q_{max}}+\frac{1}{q_{max}KC}$$

Here, *q_e_* (mg g^−1^) represents the amount of pollutant adsorbed at equilibrium, *q_max_* (mg g^−1^) is the theoretical maximum adsorption capacity, *K* (L mg^−1^) is the Langmuir constant related to the affinity of the binding sites, and *C* (mg L^−1^) is the equilibrium concentration of the pollutant in solution.

In addition, the Freundlich model is characterized by the following equation:

(2) $$q_{e}=K_{F}C_{e}^{\frac{1}{n}}$$

Here, *K_F_* (with units of mg (mg (L mg^−1^)^1/2^ g^−1^) is a constant related to bonding energy, representing the adsorption capacity of pollutant on adsorbents per unit equilibrium concentration. 1/*n* (0~1) reflects the adsorption intensity or surface heterogeneity.

For flow through adsorption, 60 mg of MNCAs acted as filtration units and were fixed in a syringe for continuous adsorption. Then, 5 mL of IDG solution (100 mg L^−1^) was flowed through the syringe with an MNCA-packed column. The flow rate was controlled with the push rod of syringe.

### 4.5. Electrochemical Characterization

Electrochemical performance assessments were conducted using traditional electrode fabrication techniques. For typical electrode preparation, active material:Ketjen Black:PVDF binder in a weight ratio of 4:4:2 was dissolved in N-methyl-2-pyrrolidone (NMP) to form a homogeneous slurry. This slurry was uniformly coated onto aluminum foil and dried under a vacuum at 60 °C for 12 h to yield IDG@MNCAs electrodes. The thickness and the mass loading of typical electrodes were 90 μm and 0.3~1 mg cm^−2^, respectively. Sequentially, CR2032 button-type cells were fabricated by incorporating a PP separator between the working electrode and a lithium metal foil. The assembly process was conducted in a high-purity argon-filled glove box to ensure an inert atmosphere. The electrolyte used was composed of 1.0 mol L^−1^ LiTFSI dissolved in a 1:1 volumetric mixture of 1,2-dimethoxyethane (DME), and 1,3-dioxolane (DOL).

Galvanostatic charge/discharge tests were recorded using a CT3002A battery testing system (LAND Electronic Co., Wuhan, China). Cyclic voltammetry (CV) measurements were performed at a scan rate of 1 mV s^−1^ using a CS2350M electrochemical workstation (CORRTEST Co., Wuhan, China). The loading amounts of IDG in IDG@MNCAs electrode and AZR in AZR@MNCAs were 1.09 × 10^−7^ and 1.24 × 10^−7^ mol cm^−2^, respectively. Prior to electrochemical impedance spectroscopy (EIS) analysis, all assembled batteries were pre-cycled 100 times. EIS data were acquired over a frequency range of 0.01 Hz to 100 kHz with an AC perturbation amplitude of 5 mV. Galvanostatic intermittent titration technique (GITT) experiments were measured on half-cells in the potential range of 1.3–3.5 V (vs. Li/Li^+^) after 5 cycles for stabilization. A galvanostatic pulse with a current of 25 mA g^−1^ was employed for a duration of 30 min, followed by a relaxation phase of 60 min at open circuit to equilibrium until the potential reached 1.3 V or 3.5 V (vs. Li/Li^+^). The Li^+^ diffusion coefficient (*D_Li^+^_*) for IDG@MNCAs was calculated using the equation below:

(3) $$D_{{Li}^{+}}=\frac{4}{\pi \tau }{\left(\frac{{V_{M}n}_{B}}{S}\right)}^{2}{\left(\frac{\Delta E_{s}}{\Delta E_{\tau }}\right)}^{2}$$

In this equation, *τ* is the pulse duration (*τ* = 600 s), and *n_B_* and *V_M_* are the molar quantity and molar volume of active material, respectively. *S* is the contact area between the active material and the electrolyte (cm^2^), approximated as the geometric area of the electrode. *ΔE_s_* is the steady-state voltage difference between two adjacent relaxation periods, and *ΔE_τ_* is the transient voltage change during each pulse.

## Data Availability

The data presented in this study are available upon request from the corresponding author.

## Supplementary Materials

The following supporting information can be downloaded at: https://www.mdpi.com/article/10.3390/gels11110857/s1, Figure S1. Raman spectrum of NPAs; Figure S2. SEM image of NSPS prepared in (a) DMF and (b) 1,2-dichloroethane; Figure S3. HRTEM image of cNPAs; Figure S4. XRD patterns of MNCAs and cNPAs; Figure S5. XPS survey spectra of samples; Figure S6. High-resolution XPS spectra of (a) C 1s and (b) O 1s and (c) N 1sfor MNCAs; Figure S7. SEM images of (a) MNCAs-g and (b) MNCAs-t; Figure S8. (a) N_2_ adsorption–desorption isotherms and (b) Pore size distribution of MNCAs-g and MNCAs-t; Figure S9. photographs (before and after adsorption) of IDG and AZR dyes; Figure S10. (a) Adsorption capacities of IDG onto MNCAs with varying IDG concentrations, (b) The Langmuir–Freundlich plots of MNCAs toward IDG; Figure S11. UV spectra of IDG adsorption under different (a) pH values, (b) temperatures, and (c) NaCl concentrations. Removal efficiency of IDG under different (d) pH values, (e) temperatures, and (f) NaCl concentrations; Figure S12. Raman spectra of MNCAs, IDG@MNCAs and AZR@MNCAs; Figure S13. CV curves at 1 mV s^−1^ of (a) MNCAs, (b) IDG, and (c) AZR; Figure S14. Digital photograph of separators stripped from homologous cells after 10 cycles; (a) discharged and (b) charged of IDG; (c) discharge and (d) charged of IDG@MNCAs; Figure S15. Nyquist plots of MNCAs; Figure S16. (a) cycling performance of AZR-IDG@MNCAs tested at 100 mA g^−1^. (b) Rate capacity test of AZR-IDG@MNCAs under different current density; Table S1. Element contents of the samples; Table S2. BET specific surface area and pore volumes of the samples; Table S3. Comparison of cycle performance between IDG@MNCAs and other organic hybrid composite cathodes; Video S1: gelation process of NPAs.

## References

[1] Lin J., Zhang X., Fan E., Chen R., Wu F., Li L. (2023). Carbon neutrality strategies for sustainable batteries: From structure, recycling, and properties to applications. Energy Environ. Sci..

[2] Wu Y., Li H., Liu T., Xu M. (2023). Versatile Protein and Its Subunit Biomolecules for Advanced Rechargeable Batteries. Adv. Mater..

[3] Shen J., Zhou M., Liu W., Shi Y., Tang W., Deng Y., Liu R., Zuo Y., Zhang J. (2025). Advanced direct recycling technology enables a second life of spent lithium-ion battery. Energy Storage Mater..

[4] Zhang J., White J.C., He J., Yu X., Yan C., Dong L., Tao S., Wang X. (2025). Sustainable bioactive hydrogels for organic contaminant elimination in wastewater. Nat. Commun..

[5] Ji X., Wang H., Wang H.Y., Zhao T., Page Z.A., Khashab N.M., Sessler J.L. (2020). Removal of Organic Micropollutants from Water by Macrocycle-Containing Covalent Polymer Networks. Angew. Chem. Int. Ed..

[6] Peydayesh M., Suter M.K., Bolisetty S., Boulos S., Handschin S., Nyström L., Mezzenga R. (2020). Amyloid Fibrils Aerogel for Sustainable Removal of Organic Contaminants from Water. Adv. Mater..

[7] Song S.H., Kim H.S., Kim K.S., Hong S., Jeon H., Lim J., Jung Y.H., Ahn H., Jang J.D., Kim M.-H. (2023). Toward a Nanoscale-Defect-Free Ni-Rich Layered Oxide Cathode Through Regulated Pore Evolution for Long-Lifespan Li Rechargeable Batteries. Adv. Funct. Mater..

[8] Lee S., Li C., Manthiram A. (2024). Effects of Calcination Conditions on the Structural and Electrochemical Behaviors of High-Nickel, Cobalt-Free LiNi_0.9_Mn_0.1_O_2_ Cathode. Adv. Energy Mater..

[9] Zuo Y., Liu J., Wang H., Zou Y., Luo T., Zhang K., Yang Y., Gao C., Li B., Sun Q. (2025). T^#^2-Li_0.69_CoO_2_: A Durable, High-Capacity, High-Rate Cathode Material for Lithium-Ion Batteries. Adv. Mater..

[10] Chen H., Yuan H., Dai Z., Feng S., Zheng M., Zheng C., Jin J., Wu M., Wu X., Lu J. (2024). Surface Gradient Ni-Rich Cathode for Li-Ion Batteries. Adv. Mater..

[11] Li W., Cheng C., Zhao J., Song Y., Xue C. (2025). Enhanced Azo Dye Removal through Sequential Ultrasound-Assisted-Treatment and Photocatalysis Using CdZnS. Angew. Chem. Int. Ed..

[12] Li C., Guggenberger P., Han S.W., Ding W.-L., Kleitz F. (2022). Ultrathin Covalent Organic Framework Anchored on Graphene for Enhanced Organic Pollutant Removal. Angew. Chem. Int. Ed..

[13] Amen R., Elsayed I., Kim Y., Schueneman G.T., El-Giar E.M., Hassan E.B. (2025). A Novel Green in Situ Amine-Functionalized Aerogel UiO-66-NH_2_/TOCNF for the Removal of Azo Anionic Dyes. Gels.

[14] Ejsmont A., Dutta S., Jankowska A., Wuttke S., Goscianska J. (2024). Governing the Porosity and Morphology of Zeolitic Imidazolate Framework-Derived Functional Carbons toward Improved Adsorption of Cationic Dyes. Chem. Mater..

[15] Sui Q., Huang J., Liu Y., Chang X., Ji G., Deng S., Xie T., Yu G. (2011). Rapid removal of bisphenol A on highly ordered mesoporous carbon. J. Environ. Sci..

[16] Yang H., Lee J., Cheong J.Y., Wang Y., Duan G., Hou H., Jiang S., Kim I.-D. (2021). Molecular engineering of carbonyl organic electrodes for rechargeable metal-ion batteries: Fundamentals, recent advances, and challenges. Energy Environ. Sci..

[17] Gu S., Chen J., Hussain I., Wang Z., Chen X., Ahmad M., Feng S.-P., Lu Z., Zhang K. (2024). Modulation of Radical Intermediates in Rechargeable Organic Batteries. Adv. Mater..

[18] Lee J., Kim C., Cheong J.Y., Kim I.-D. (2022). An angstrom-level d-spacing control of graphite oxide using organofillers for high-rate lithium storage. Chem.

[19] Shi T., Li G., Han Y., Gao Y., Wang F., Hu Z., Cai T., Chu J., Song Z. (2022). Oxidized indanthrone as a cost-effective and high-performance organic cathode material for rechargeable lithium batteries. Energy Storage Mater..

[20] Zhao B., Si Y., Guo W., Fu Y. (2022). Insoluble Naphthoquinone-Derived Molecular Cathode for High-Performance Lithium Organic Battery. Adv. Funct. Mater..

[21] Luo Z., Liu L., Zhao Q., Li F., Chen J. (2017). An Insoluble Benzoquinone-Based Organic Cathode for Use in Rechargeable Lithium-Ion Batteries. Angew. Chem. Int. Ed..

[22] Yang J., Wang Z., Shi Y., Sun P., Xu Y. (2020). Poorly Soluble 2,6-Dimethoxy-9,10-anthraquinone Cathode for Lithium-Ion Batteries: The Role of Electrolyte Concentration. ACS Appl. Mater. Interfaces.

[23] He W., Cao J., Zhou X., Zhang N., Qi Y., Li J., Wu N., Liu X. (2025). Bi-Interfacial Electron Modulation in Co_9_S_8_/FeCoS_2_ Heterostructures Anchored on Bamboo-Derived Carbon Quasi-Aerogel for High-Performance Hydrogen Evolution. Gels.

[24] Chen Z., Liu S., Chen L., Huang J., Zheng B., Huang W., Li S., Lu Y., Fu R. (2021). A scalable molecular-templating strategy toward well-defined microporous carbon aerogels for efficient water treatment and electrocatalysis. Chem. Eng. J..

[25] Chen Z., Liu S., Huang J., Huang W., Chen L., Cui Y., Du Y., Fu R. (2021). Molecular Level Design of Nitrogen-Doped Well-Defined Microporous Carbon Spheres for Selective Adsorption and Electrocatalysis. ACS Appl. Mater. Interfaces.

[26] Wang J., Xu Z., Eloi J.-C., Titirici M.-M., Eichhorn S.J. (2022). Ice-Templated, Sustainable Carbon Aerogels with Hierarchically Tailored Channels for Sodium- and Potassium-Ion Batteries. Adv. Funct. Mater..

[27] Liu J., Yang F., Cao L., Li B., Yuan K., Lei S., Hu W. (2019). A Robust Nonvolatile Resistive Memory Device Based on a Freestanding Ultrathin 2D Imine Polymer Film. Adv. Mater..

[28] Chen Z., Xiong Y., Liu Y., Wang Z., Zhang B., Liang X., Chen X., Yin Y. (2024). Precisely Designed Morphology and Surface Chemical Structure of Fe-N-C Electrocatalysts for Enhanced Oxygen Reaction Reduction Activity. Molecules.

[29] Yu Z.-L., Yang N., Apostolopoulou-Kalkavoura V., Qin B., Ma Z.-Y., Xing W.-Y., Qiao C., Bergström L., Antonietti M., Yu S.-H. (2018). Fire-Retardant and Thermally Insulating Phenolic-Silica Aerogels. Angew. Chem. Int. Ed..

[30] Wang M., Wang W., Qian T., Liu S., Li Y., Hou Z., Goodenough J.B., Ajayan P.M., Yan C. (2019). Oxidizing Vacancies in Nitrogen-Doped Carbon Enhance Air-Cathode Activity. Adv. Mater..

[31] Zhang M., Hu M., Liu X., Zhang Y., Yuan X., Zhang Z., Xiong K., Zhang W., Xu Q., Ren Z. (2026). Tailoring heteroatom-doped porous carbon materials for efficient adsorption of volatile organic compounds: Fabrication strategies, doping effects, and adsorption mechanisms. Coord. Chem. Rev..

[32] Wang Z., Li Y., Liu P., Qi Q., Zhang F., Lu G., Zhao X., Huang X. (2019). Few layer covalent organic frameworks with graphene sheets as cathode materials for lithium-ion batteries. Nanoscale.

[33] Fu M., Zhang C., Chen Y., Fan K., Zhang G., Zou J., Gao Y., Dai H., Wang X., Wang C. (2022). A thianthrene-based small molecule as a high-potential cathode for lithium-organic batteries. Chem. Commun..

[34] Dai G., He Y., Niu Z., He P., Zhang C., Zhao Y., Zhang X., Zhou H. (2019). A Dual-Ion Organic Symmetric Battery Constructed from Phenazine-Based Artificial Bipolar Molecules. Angew. Chem. Int. Ed..

[35] Xu L., Wang G., Yao L., Su C. (2024). Molecular Design Strategy toward Multielectron-Based Polyphenylaniline Organic Cathode and Its Electrochemical Performance. ACS Appl. Energy Mater..

[36] Lee K., Serdiuk I.E., Kwon G., Min D.J., Kang K., Park S.Y., Kwon J.E. (2020). Phenoxazine as a high-voltage p-type redox center for organic battery cathode materials: Small structural reorganization for faster charging and narrow operating voltage. Energy Environ. Sci..

[37] Gong Y., Zhang W., Liu Z., Fang M., Yang J., Wang Y., Gao M., Zhang J., Yang Q.-H., Li Z. (2024). Phenothiazine Derivatives as Small-Molecule Organic Cathodes with Adjustable Dropout Voltage and Cycle Performance. Adv. Mater..

[38] Bhosale M., Schmidt C., Penert P., Studer G., Esser B. (2024). Anion-Rocking Chair Batteries with Tuneable Voltage using Viologen- and Phenothiazine Polymer-based Electrodes. ChemSusChem.

[39] Lap T., Goujon N., Mantione D., Ruipérez F., Mecerreyes D. (2023). Bio-Based Polyhydroxyanthraquinones as High-Voltage Organic Electrode Materials for Batteries. ACS Appl. Poly. Mater..

[40] Wu N., Shen J., Li Q., Li S., Guo D., Li J., Liu G., Zhao J., Cao A., Mi H. (2025). Synergistic Bimetallic Interaction and Regulated Void Size in Isocubanite CuFe_2_S_3_ Enables Ultra Fast and Durable Sodium Storage. ACS Sustain. Chem. Eng..

[41] Li H., Li X., Liu J., Ding N.W., Zhang Q., Lei S., Sydorov D. (2025). Synergistically Electronic and Structural Regulation of VO_2_ by Ce Doping Toward High-Performance Zinc-Ion Batteries. ACS Sustain. Chem. Eng..

