# Supplementary material for: Rational Design of N-Doped Carbon Aerogel with Well-Defined Micropore Structure to Adsorb Dye from Water for High-Performance Lithium-Ion Battery Cathodes
Source: Gels. 2025 Oct 27;11(11):857. doi: 10.3390/gels11110857 (PMC12652416; doi:10.3390/gels11110857)
Supplement: Supplementary file 1 [file gels-11-00857-s001.zip › gels-3930898-supplementary .pdf]

Supporting Information

Rational design of N-doped carbon aerogel with well-defined micropore structure to adsorb dye from water for high-performance Lithium-ion battery cathodes

Yuang Xiong, Kelin Zhu, Lixia Yang, Rong Huang,Xingtang Liang, Binbin Zhang, Yanzhen Yin, Xia Chen and Zirun Chen*

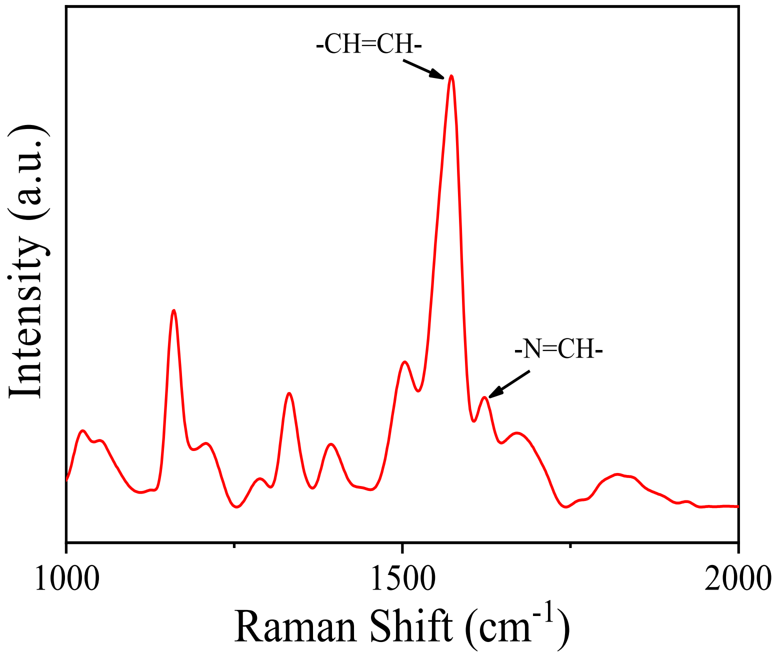

Figure S1. Raman spectrum of NPAs.

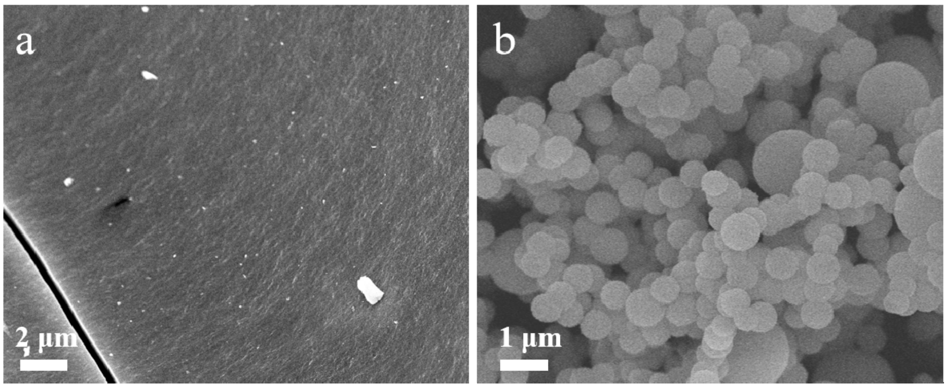

Figure S2. SEM image of NSPS prepared in (a) DMF and (b) 1,2-dichloroethane.

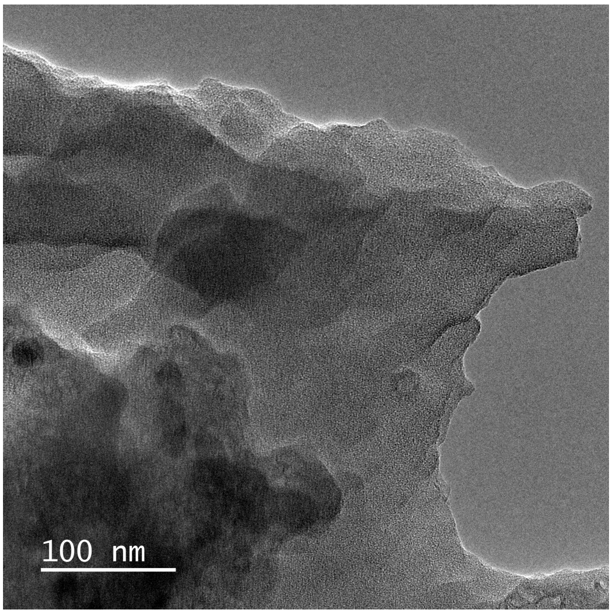

Figure S3. HRTEM image of cNPAs.

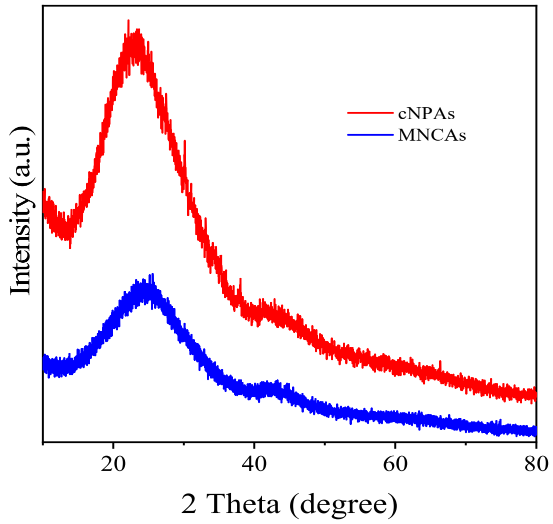

Figure S4. XRD patterns of MNCAs and cNPAs.

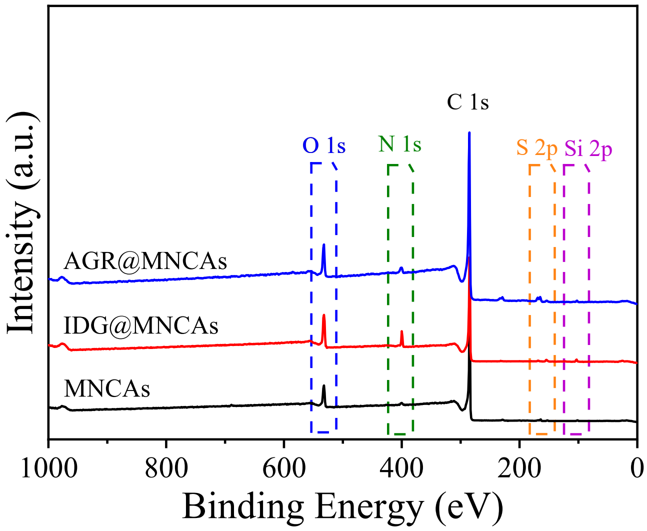

Figure S5. XPS survey spectra of samples.

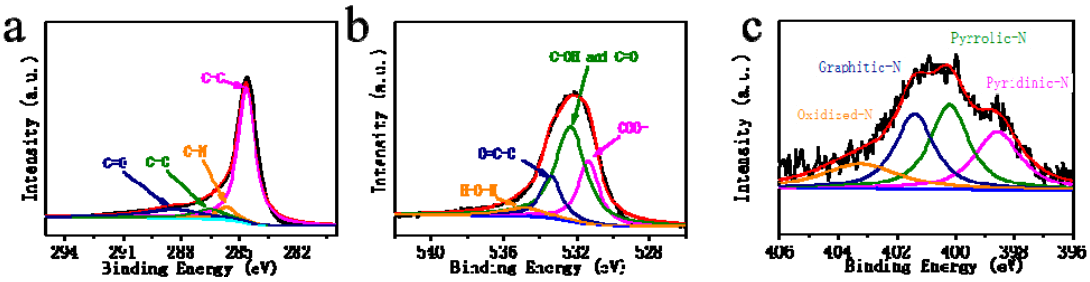

Figure S6. High resolution XPS spectra of (a) C 1s and (b) O 1s and (c) N 1sfor MNCAs.

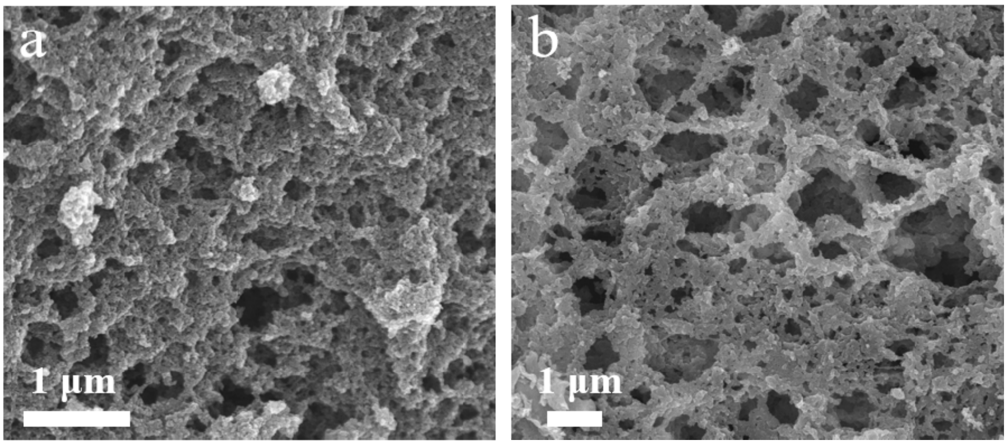

Figure S7. SEM images of (a) MNCAs-g and (b) MNCAs-t.

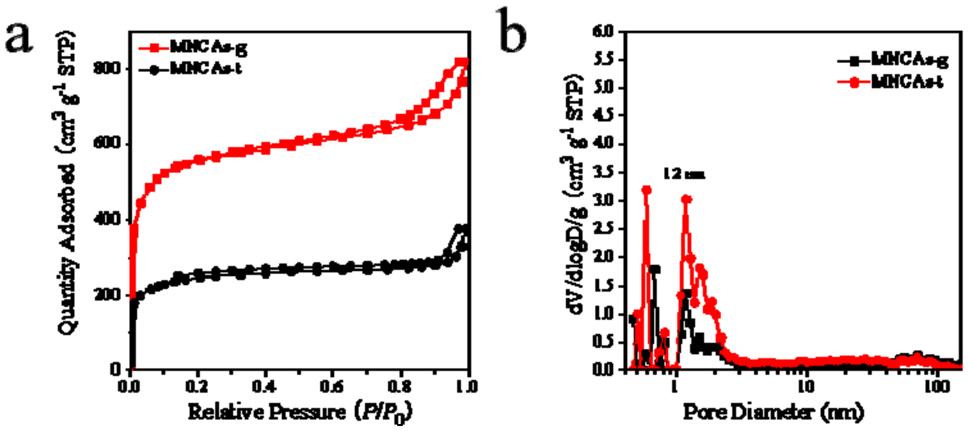

Figure S8. (a) N_2 adsorption–desorption isotherms and (b) Pore size distribution of MNCAs-g and MNCAs-t

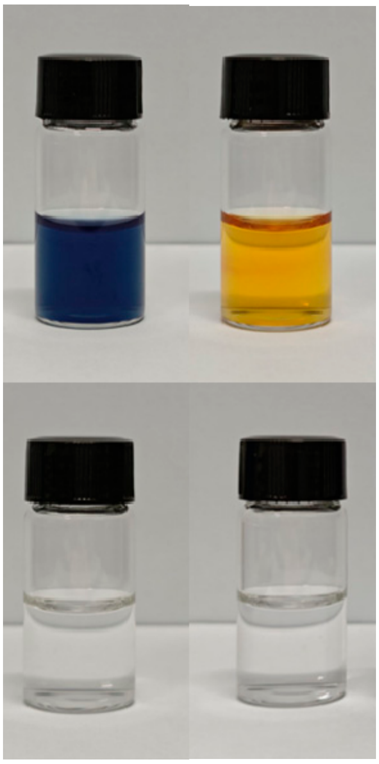

Figure S9. photographs (before and after adsorption) of IDG and AZR dyes.

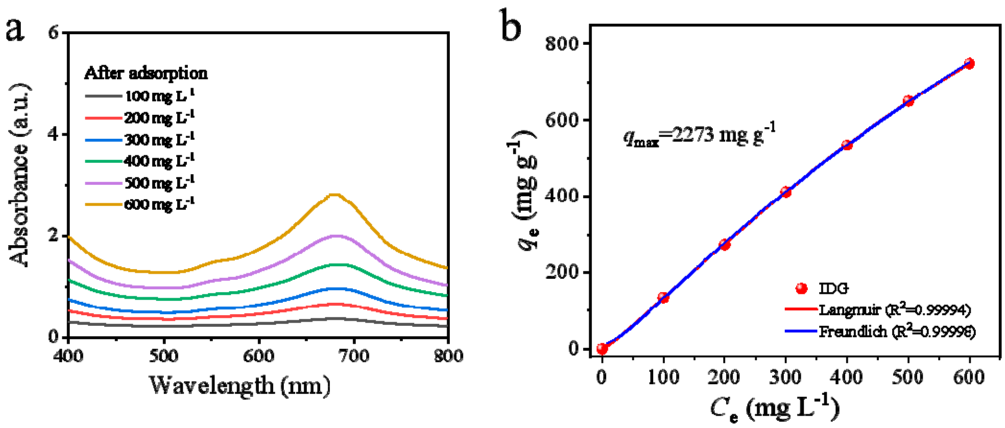

Figure S10. (a) Adsorption capacities of IDG onto MNCAs with varying IDG concentrations, (b) The Langmuir-Freundlich plots of MNCAs toward IDG.

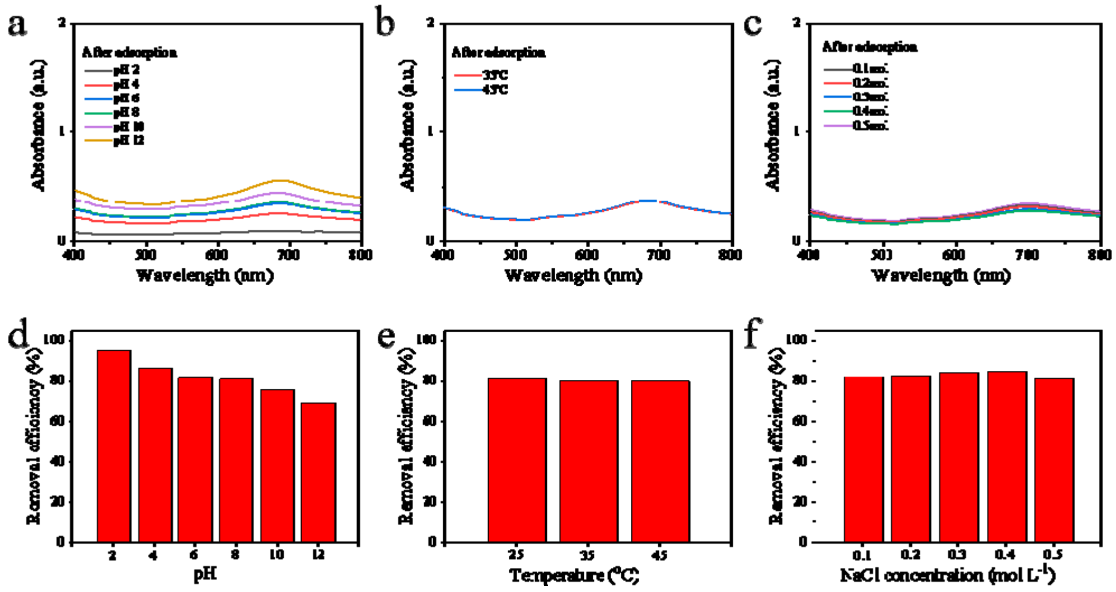

Figure S11. UV-vis spectra of IDG adsorption under different (a) pH values; (b) temperatures and (c) NaCl concentratios. Removal efficiency of IDG uder different (d) pH values; (e) temperatures and (f) NaCl concentratios.

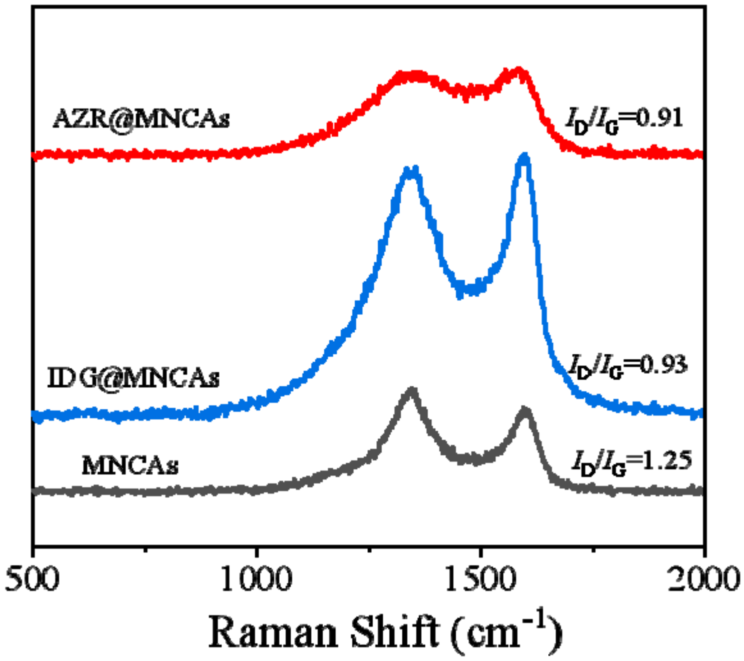

Figure S12. Raman spectra of MNCAs, IDG@MNCAs and AZR@MNCAs.

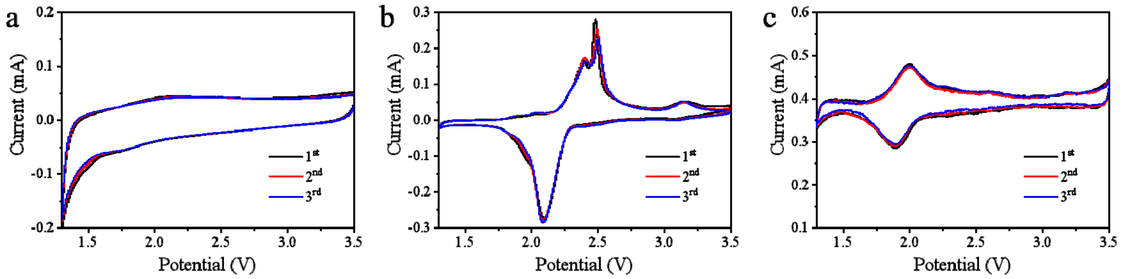

Figure S13. CV curves at 1 mV s^{-1} of (a) MNCAs, (b) IDG and (c) AZR.

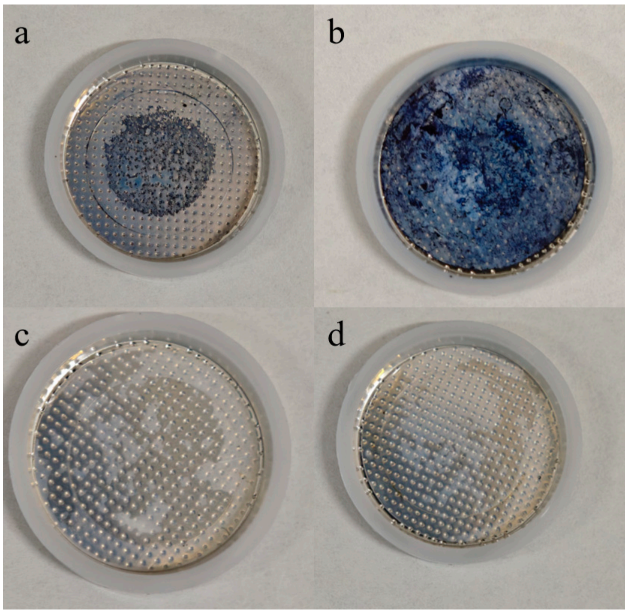

Figure S14. Digital photograph of separators stripped from homologous cells after 10 cycles; (a) discharged and (b) charged of IDG; (c) discharge and (d) charged of IDG@MNCAs.

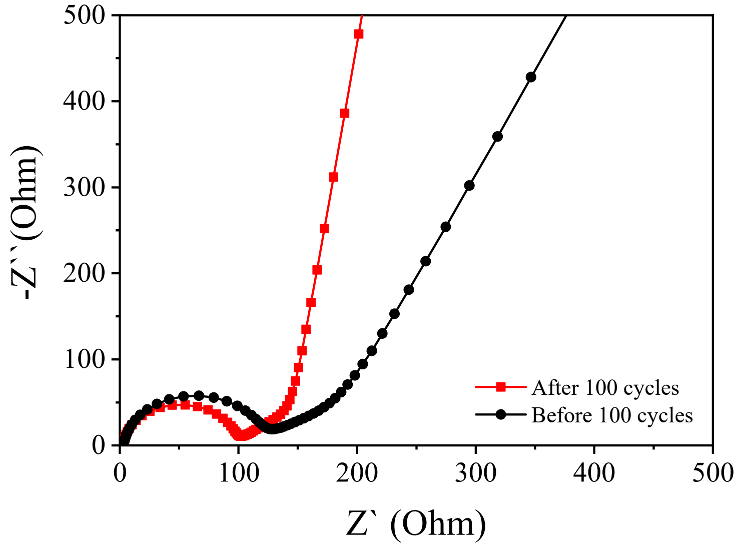

Figure S15. Nyquist plots of MNCAs.

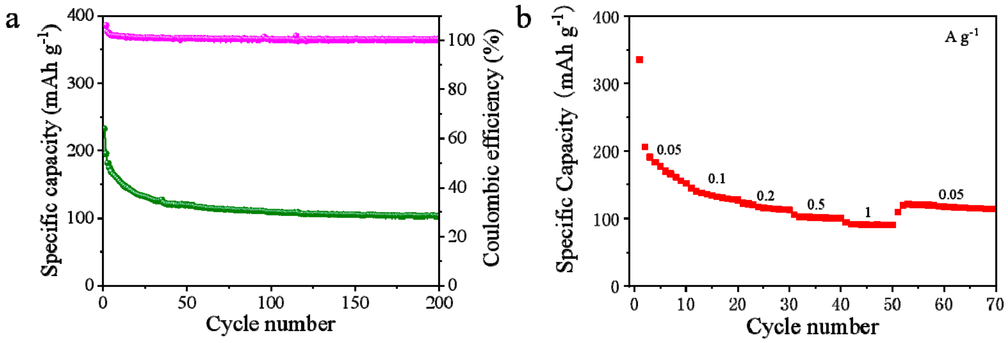

Figure S16. (a) cycling performance of AZR-IDG@MNCAs tested at 100 mA g^{-1}. (b) Rate capacity test of AZR-IDG@MNCAs under different current density.

Table S1. Element contents of the samples.

Samples	C content (at%)	N content (at%)	O content (at%)	Si content (at%)	S content (at%)
MNCAs	88.54	1.69	9.17	0.6	/
IDG@MNCAs	78.2	6.53	13.59	1.68	/
AZR@MNCAs	76.43	5.58	16.62	0.64	0.72

Table S2. BET specific surface area and pore volumes of the samples.

Samples	S_{BET} (m^2 g^{-1})	$S_{micropore}$ (m^2 g^{-1})	$S_{mesopore}$ (m^2 g^{-1})	Pore volume (cm^3 g^{-1})
cNPAs	789	663	126	0.51
MNCAs	1986	1262	724	1.06
MNCAs-g	847	500	347	0.58
MNCAs-t	1914	1272	642	1.27

Table S3. Comparison of cycle performance between IDG@MNCAs and other organic hybrid composite cathodes.

Sample	Capacity retention (mAh g^{-1}), cycle number, current density (mA g^{-1}), retention	Reference
IDG@MNCAs	120, 200, 100, 89%<br>76, 1200, 1000, 70%	This work
COF/reduced graphene composite	67,300,128, 54%	1
Thianthrene-based small molecule	63,100,500, 86%	2
Phenazine-based small molecule	96,100,124, 91%	3
p-PhPTZOMe	111,100,20, 83%	4
Phenoxazine/active carbon composite	74,500,120, 80%	5
Phenothiazine derivatives	59,1000,104, 60%	*6*
Phenothiazine-based polymer	60,50,11.2, 80%	*7*
Polyhydroxyanthraquinones/MWCNTs composite	82,100,591, 51%	8

1. Wang, Z.; Li, Y.; Liu, P.; Qi, Q.; Zhang, F.; Lu, G.; Zhao, X.; Huang, X. Few layer covalent organic frameworks with graphene sheets as cathode materials for lithium-ion batteries. *Nanoscale* **2019**, *11*, 5330-5335.

2. Fu, M.; Zhang, C.; Chen, Y.; Fan, K.; Zhang, G.; Zou, J.; Gao, Y.; Dai, H.; Wang, X.; Wang, C., A thianthrene-based small molecule as a high-potential cathode for lithium–organic batteries. *Chem. Commun.* **2022**, *58*, 11993-11996.

3. Dai, G.; He, Y.; Niu, Z.; He, P.; Zhang, C.; Zhao, Y.; Zhang, X.; Zhou, H. A Dual-Ion Organic Symmetric Battery Constructed from Phenazine-Based Artificial Bipolar Molecules. *Angew. Chem. Int. Ed.* **2019**, *58*, 9902-9906.

4. Xu, L.; Wang, G.; Yao, L.; Su, C., Molecular Design Strategy toward Multielectron-Based Polyphenylaniline Organic Cathode and Its Electrochemical Performance. *ACS Appl. Energy Mater.* **2024**, *7*, 8445-8454.

5. Lee, K.; Serdiuk, I. E.; Kwon, G.; Min, D. J.; Kang, K.; Park, S. Y.; Kwon, J. E. Phenoxazine as a high-voltage p-type redox center for organic battery cathode materials: small structural reorganization for faster charging and narrow operating voltage. *Energy Environ. Sci.* **2020**, *13*, 4142-4156.

6. Gong, Y.; Zhang, W.; Liu, Z.; Fang, M.; Yang, J.; Wang, Y.; Gao, M.; Zhang, J.; Yang, Q.-H.; Li, Z. Phenothiazine Derivatives as Small-Molecule Organic Cathodes with Adjustable Dropout Voltage and Cycle Performance. *Adv. Mater.* **2024**, *36*, 2312486.

7. Bhosale, M.; Schmidt, C.; Penert, P.; Studer, G.; Esser, B. Anion-Rocking Chair Batteries with Tuneable Voltage using Viologen- and Phenothiazine Polymer-based Electrodes. *ChemSusChem* **2024**, *17*, 202301143.

8. Lap, T.; Goujon, N.; Mantione, D.; Ruipérez, F.; Mecerreyes, D. Bio-Based Polyhydroxyanthraquinones as High-Voltage Organic Electrode Materials for Batteries. *ACS Appl. Poly. Mater.* **2023**, *5*, 9128-9137.
